# Overcoming barriers to single-cell RNA sequencing adoption in low- and middle-income countries

**DOI:** 10.1038/s41431-024-01564-4

**Published:** 2024-04-02

**Authors:** Tracy Boakye Serebour, Adam P. Cribbs, Mathew J. Baldwin, Collen Masimirembwa, Zedias Chikwambi, Angeliki Kerasidou, Sarah J. B. Snelling

**Affiliations:** 1 The Botnar Institute for Musculoskeletal Science, Nuffield Department of Orthopaedics, Rheumatology and Musculoskeletal Sciences, University of Oxford, Oxford, UK; 2 The Ethox Centre and the Wellcome Centre for Ethics and Humanities, Nuffield Department of Population Health, University of Oxford, Oxford, UK; 3 The African Institute of Biomedical Science and Technology, Harare, Zimbabwe

**Keywords:** Ethics, RNA sequencing, Sequence annotation

## Abstract

The advent of single-cell resolution sequencing and spatial transcriptomics has enabled the delivery of cellular and molecular atlases of tissues and organs, providing new insights into tissue health and disease. However, if the full potential of these technologies is to be equitably realised, ancestrally inclusivity is paramount. Such a goal requires greater inclusion of both researchers and donors in low- and middle-income countries (LMICs). In this perspective, we describe the current landscape of ancestral inclusivity in genomic and single-cell transcriptomic studies. We discuss the collaborative efforts needed to scale the barriers to establishing, expanding, and adopting single-cell sequencing research in LMICs and to enable globally impactful outcomes of these technologies.

## Introduction

High-throughput genomic methods have revolutionised biomedical sciences, making critical contributions to recent healthcare advancements [1]. Examples include improved understanding of gene mutations, genetic testing, and pharmacogenetic therapy through DNA- and, more recently, single-cell resolution RNA-sequencing analysis. These advancements have helped to clarify the role of genetic and corresponding cellular factors in disease pathogenesis.

The Human Genome Project (HGP), completed in 2003, was a landmark scientific achievement that pioneered a global scientific collaboration to generate a representative human genome sequence [2]. The cost of genomic sequencing per base has dramatically decreased to just 0.0014% of its price in 2001, enabling its increasing adoption across global settings [3]. Several DNA- and disease-focused consortia were subsequently founded, including The International Haplotype Mapping (HapMap) [4], The 1000 Genomes Project [5], The Cancer Genome Atlas (TCGA) [6], The MalariaGEN [7], and The Human Pangenome Project (HPP) [8].

However, it is the temporal control of the transcription of genes, or combination of genes, within specific cell states or types that provides the basis for development, homoeostasis and pathology. The recent rapid development of laboratory and computational approaches for RNA analysis [9], such as single-cell and spatial transcriptomics, has now enabled the additional analysis of cellular-level transcriptomics.

Single-cell resolution RNA-seq (scRNAseq) can define cellular heterogeneity (e.g. the cell types or cell states) within an organ, tissue or biological sample by providing a transcriptomic signature for each cell present [10]. ScRNAseq can therefore be used to investigate the impacts of factors including genetic variants, drug treatments and environmental factors on the cellular basis of tissue development, health, and disease [11]. For maximal pharmacogenomics and biomedical impact, ancestral diversity, which is largely missing from the HGP [12], must be included—to, for example, enable understanding of complex population traits such as drug response variation and susceptibility to common or rare diseases.

This article explores the historical under-representation of global populations in genomics and explores approaches to achieve ancestral diversity in genomics as well as single-cell transcriptomics. We also discuss the ambiguous meaning of ‘representation’ in biomedical research and current efforts aiming to address persistent underrepresentation. Finally, we highlight efforts to construct reference cell atlases and the challenges in establishing single-cell RNA sequencing capacities to deliver equitable and globally relevant diverse atlases in LMICs. Box 1.

Box 1 The meaning and interpretation of representation in genomic research The meaning of ancestral representation is often ambiguous and inconsistent, without clear definitions there is a risk of losing the meaning of diversity [16, 67]. The principle of representation is often understood as either: (1) “capturing genetic variation” or (2) “an ethical imperative to include marginalized social groups with a disproportionate burden of disease” [68]. While often perceived to result in the same outcomes, these aims are distinct. In short, filling genetic gaps does not automatically guarantee the active inclusion of historically ignored communities in its scientific translation [69]—which itself influences their hesitancy to participate and perpetuates the problem [68, 69].

Instead, when operationalising the meaning of representation, researchers should align the complementary aims of capturing genetic variation whilst simultaneously redressing health inequities. Put another way, a project’s success should be measured by its impact on reducing health disparities, in addition to filling in the gaps in ancestral information [21, 70].

## The history of underrepresentation within the genomic reference sequence and its current implications

The Human Genome Reference Sequence from the HGP project is the accepted reference for scientists to compare their DNA sequencing results and helps promote genomic data sharing [13]. The HGP project was an international collaboration. However, several global regions like Africa, East Asia, South America, and The Middle East were underrepresented in the genomics reference sequence [2, 8]. The current human reference (GRCh38.p13), revised in 2019 by the Genome Reference Consortium, lacks ancestral diversity and comprehensiveness that reflects the genetic variation amongst humans—compiled using sequencing data from approximately 20 individuals who were predominantly of European descent [2, 13]. However, approximately 70% of this data came from an individual of European descent with a high risk for diabetes [9, 14], so it does not represent a “healthy genome” or a diverse collection of high-quality reference haplotypes [15]. This reference framework’s limitations result in biases, incomplete data, inaccurate genetic analysis, and lack of genetic variation.

This inequity in benefit of the HGP has been further compounded by the fact that most subsequent large-scale human genetic studies have focused on populations of European ancestry [16]. This global underrepresentation contributes to the knowledge gap in the genetic factors influencing tissue health, disease inheritance, drug efficacy, and response [12]. Mapping genomic diversity could provide insight into novel genetic and epigenetic signals that influence disease development and enable personalised treatment outcomes for diverse patient populations [17].

The genomic reference sequence does however continue to undergo changes to provide a more robust and representative panel [18]. In response to the lack of diversity in the HGP, the International HapMap project (2002–2010) and the 1000 Genomes Project (2008-2015) were established to provide a more comprehensive and diverse representation of the human genome [16, 19]. Ancestral diversity within their reference panels represents major populations from Europe, West Africa, South and East Asia and the Americas [20]

While there is now a universal recognition that promoting genomic research in diverse populations is essential to delivering equitable healthcare [21], logistical, ethical, and technological setbacks have ensued [22]. Drawing lessons from the shortcomings of the HGP and the challenges encountered is relevant to the delivery of ancestrally inclusive single-cell transcriptomic atlases.

## Efforts to generate an ancestrally representative human genomic reference

The Human Pangenome Project (HPP), funded by the National Genome Research Institute (NHGRI), aims to assess the current gaps in diversity to correct the biases and errors within the existing human reference genome framework, creating a more complete and accurate representation of the genomic blueprint of the human species [8, 13]. The project and population-specific ones like it, including The African Genome Variation Project [23], The Greater Middle East Variome Project [24], The Latin American Genomics Consortium [25], The National Centre for Indigenous Genomics in Australia [26] and more aim to overcome the challenges of performing representative genomic research. The recently completed first complete sequence of the haploid human genome (T2T-CHM13) by the Telomere-to-Telomere (T2T) consortium has provided a template for enhancing genomic analyses and the construction of reference genomes from ancestrally diverse populations [27, 28]. This comprehensive sequence improves more precise read mapping and variant identification in globally diverse populations, refining the understanding of human genetic variation and disease-related variants at the DNA level [28]. This enables the scientists collaborating on the HPP to capture global genetic variation from 350 ancestrally diverse people to generate the total or missing sequences with improved DNA sequencing technologies [29]. This is to combine and create a unified genomic representation of the human species, calling it the “pangenome” [8, 29]. A representative and high-quality transcriptomic reference cell atlas of the tissues and organs of the human body will reciprocally complement and add further utility to pangenome efforts.

## Building a representative human cell atlas

The last decade has seen phenomenal efforts in transcriptomic profiling of cells, tissues and organs, leading to breakthroughs in understanding the cellular basis of common and rare diseases [30]. Current efforts to provide a transcriptomic reference dataset of the human body at single-cell resolution are in congruence with today’s pressing medical needs [31].

**Fig. 1:**
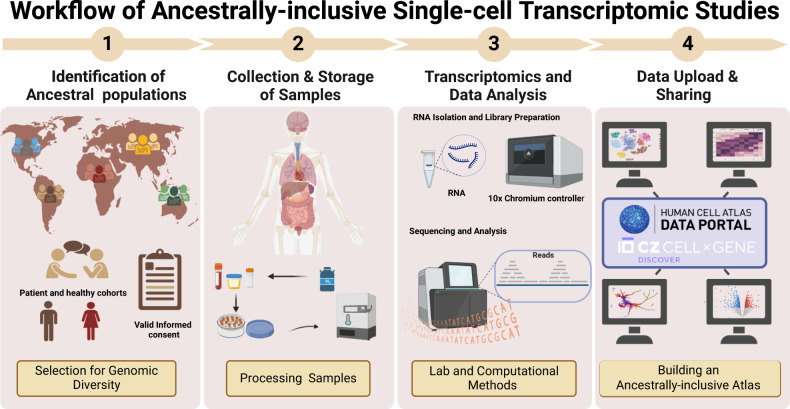
Workflow for ancestrally diverse single-cell transcriptomic and spatial omics studies in low- and middle-income countries. Step 1: Ancestral populations in low- and middle-income countries are identified. Pre-study administrative work is done to define how the study conceptualises diversity to inform the selection of cohorts. Then, engagement with intended populations contributes to establishing appropriate bioethical frameworks, emphasising location-specific valid informed consent protocols. Step 2: Tissue samples are collected and processed promptly according to tissue type and selected processing method. Patient and tissue sample processing metadata is documented. Consistency in sample selection and processing is key to enable integration of patient data in downstream analysis and mitigation of the effect of technical variation, respectively. Step 3: RNA is isolated from the sample and undergoes library preparation and sequencing to visualise gene expression. If working across multiple sites, it is important to standardise laboratory methods for quality control. Step 4: Data processing, normalising, visualising, annotating, and integration occur locally. All published datasets are made available on the Human Cell Atlas Data portal and the Chan Zuckerberg CELLxGENE Discover Portal. All code used or developed is deposited to platforms like GitHub as part of efforts to build an open-source and ancestrally inclusive reference cell atlas.

Creating a “reference atlas” of cell types and cell states within the human body is a goal of international single-cell initiatives, such as the Human Cell Atlas (HCA) [32] and the Human BioMolecular Atlas Program (HuBMAP) [33]. These projects aim to develop a widely accessible atlas of the human body at a single-cell resolution by building rich biobank resources and developing robust tissue processing and data acquisition protocols and spatial mapping algorithms [34]. Through the early inclusion of under-represented populations, these projects hope to break a cycle of minimal scientific inclusion, especially as they carry a significant proportion of the global disease burden ref [35] [Fig. 1]. However, undertaking single-cell transcriptomic studies in LMICs comes with specific infrastructural, technical, and ethical challenges.

## Infrastructural challenges

### Limited institutional capacity

The orchestration of multidisciplinary teams remains pivotal for the seamless production of high-quality single-cell data. Nevertheless, this task is marred by its inherent challenges, particularly in the context of the difficulty of working in under-resourced regions. This can be daunting due to the limited number of individuals with training and proficiency in laboratory protocols for scRNASeq. A relatively high turnover of individuals with such expertise seeking better prospects to advance their scientific careers understandably presents difficulties sustaining long-term research studies. Furthermore, it is worth emphasising that high-quality ScRNASeq, spatial transcriptomic and associated validation studies are equipment-heavy, requiring specialised instruments that often include microfluidic or plate-based devices for library preparation, alongside requirements for items including fluorescent microscopes, flow cytometers and computational hardware.

The inability to establish local core research facilities that support scRNAseq studies not only diminishes the sense of ownership in research but also impedes the advancement and full participation of scientists in LMICs in ambitious projects, including constructing a Human Cell Atlas. Addressing these issues by investing in local research infrastructure, offering training, and supporting scientists keen on conducting single-cell transcriptomic studies in LMICs could promote greater independence, increase research output, and enhance the overall success of research studies. Specifically, training involving research skills development on the undergraduate, postgraduate, and postdoctoral levels, along with continuous professional development in single-cell laboratory techniques, data analyses, and grant rewriting could be a paradigm shift in building institutional capacity for conducting single-cell transcriptomic studies [36]. Toward securing essential core facilities, access to sustainable funding from both international and local entities is critical to overcome these barriers and establish sustainable research infrastructure for transcriptomic studies to spur scientific progress in these regions.

### Unreliable power supply and lack of effective laboratory equipment maintenance

An inconsistent power supply is a significant issue affecting laboratory and computational workflows as well as the tissue storage facilities, critical for preserving the RNA integrity of stored tissues and reagents. While most facilities have alternate power sources such as generators, running a centre on backup generators during power outages can be prohibitively expensive. Additionally, even low-maintenance solar panels may be unreliable during winter and rainy seasons [37]. Erratic power supply also plays a vital role in the breakdown of laboratory equipment due to damage from unstable currents.

**Fig. 2:**
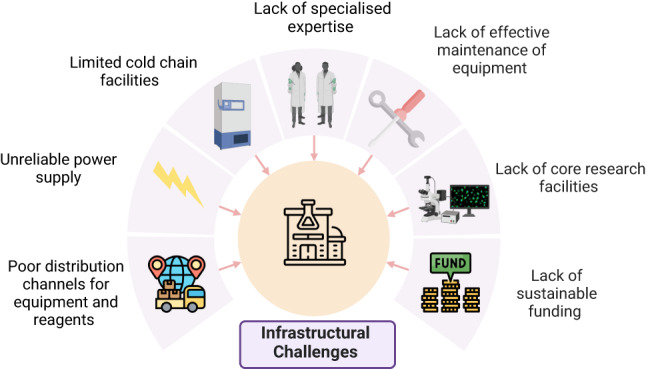
Summary of infrastructural challenges. These challenges contribute to limited Institutional capacity to conduct single-cell transcriptomic studies in low- and middle-income countries.

Moreover, the lack of resident engineers to service laboratory equipment when it breaks down, and high repair costs can greatly hinder research centres’ ability to conduct studies. For instance, in some regions, only a few technicians may be capable of servicing specialised equipment like micro-fluidic devices such as 10X Chromium^TM^ controllers, Nadia^TM^ instruments and/or devices for plate-based approaches like Smart seq2, CelSeq and MARS-seq, Next generation sequencers (NGS) (e.g. Illumina, PacBio and Oxford Nanopore single-cell sequencing platforms), NGS quantification and quality control devices (e.g. NanoDrop spectrophotometers, Qubit fluorometer, Bioanalyzer and TapeStation systems, microscopes, flow cytometers), cold storage equipment (–80 ˚C freezers), servers and more. The limited experienced service personnel present a significant challenge to the maintenance and repair of equipment [Fig. 2]. The resulting long periods of equipment inactivity and underutilisation can also reduce productivity, leading to demotivation among researchers and failure to meet project milestones.

### Sparse funding and price gouging

Scientists in LMICs experience significant barriers to accessing sustainable funding for research, and the high costs of scRNAseq studies are particularly challenging [Fig. 2] ref. [38]. Limited funding opportunities are partly due to an inadequate urgency to encourage more investment in LMICs by the private sector and local and global governments. Consequently, this delayed inaction stalls the adoption and expansion of scRNAseq technologies to move toward the level of progress high-income countries have achieved.

LMICs face substantial obstacles when procuring equipment and reagents for single-cell research due to quotations often given at prices higher than in countries more represented in biomedical science. The steep pricing is particularly burdensome for labs with limited funding, impeding their ability to competitively bid for and complete research utilising scRNAseq. While price gouging is a broader issue in conducting research within LMICs, it is especially acute in the field of transcriptomics, due to the inherent high cost of reagents and equipment prior to any pricing uplifts. LMICs are often at a disadvantage in the supply and demand chain; for instance, the market is often dominated by a limited number of companies, resulting in less price competitiveness. These market dynamics affect the sales and distribution of reagents and scRNAseq equipment within these regions, hindering the affordability of scientific research. Additionally, import restrictions and high tariffs locally and internationally further complicate the purchase and distribution of the necessary research equipment. This unavailability of reasonably priced equipment and reagents, and the complexity of distribution channels lead to the continual shipping of samples out of the country for sequencing. This, in turn, perpetuates a cycle of dependence on international collaborators to provide reagents and equipment for transcriptomic studies, or act as non-local processing sites, which establishes unequitable partnerships and stifles the development and education of new local researchers in these regions.

## Technical challenges

Regarding competence in analysing and interpreting scRNA-Seq experiments, human resources and computational, laboratory and biological knowledge are fundamental to effectively leveraging single-cell technology.

## Computational methods

**Fig. 3:**
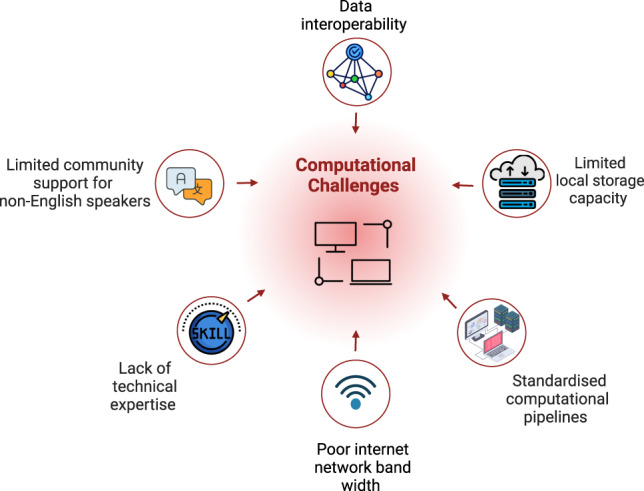
Summary of technical-computational challenges. These challenges hinder local sequencing and analysis by research groups in low- and middle-income countries.

Limited training opportunities and educational programs in genomics, transcriptomics and bioinformatics in LMICs further compound the challenges of delivering scRNA-seq datasets. Even with the ability to analyse data, the computing power needed is often suboptimal in LMICs. ScRNA-Seq generates complex and voluminous datasets that require intensive computing power for analysis. The immense computational demands of scRNA-Seq include either local- or cloud-based data storage capacities, computer clusters, and secure data management and transmission systems that are not readily available in under-resourced labs in LMICs. Moreover, additional barriers in computational analysis are due to inadequate and inconsistent internet connectivity, slow bandwidth, and a scarcity of either local storage- or cloud-based computing resources. These factors severely impede the utilisation of existing computational pipelines and software tools. In addition, harmonising computational workflows remains an issue as the field seeks to standardise processes across various platforms. The efficient analysis, modelling, and visualisation of single-cell RNA-Seq data are particularly challenged by the complexities of unifying associated metadata, ensuring data interoperability, coordinating various annotation approaches, and providing community multi-language support for non-English speakers regarding developing and troubleshooting code, among others [Fig. 3].

Fortunately, the field of computational biology is incredibly fast-paced with the advancement of new revolutionary methodologies [39, 40]. Enhancing and harmonising current computational pipelines, as well as creating cost-effective tools such as cloud-based single-cell analysis, user-friendly interfaces, and minimal hardware requirements [41, 42] can improve cost-efficiency, data storage and security, eliminate dependence on extensive IT support and maintenance as well as democratise access to computational tools that will level the playing field for research. These critical steps will serve scientists worldwide, particularly those in LMICs. These improvements are essential to facilitate efficient data capture, perform corresponding analyses, and produce interactive maps, which are critically needed.

## Laboratory methods

**Fig. 4:**
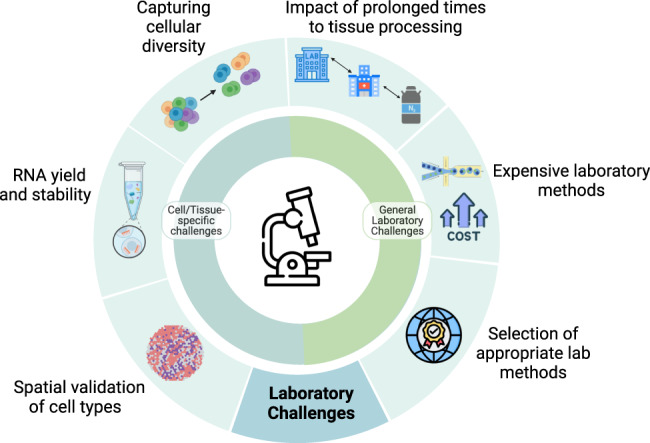
The summary of technical-laboratory challenges. Though these challenges are prevalent globally, they particularly hinder under-resourced research centres from conducting in-house single-cell experiments.

The stability of RNA and generation of unbiased data that represents biological “ground truth” is paramount in conducting single-cell studies. Therefore, the availability of core research facilities that can efficiently process and store tissue and/or cDNA libraries prior to sequencing is crucial to a study’s success. Delays between tissue collection at hospitals and enzymatic digestion to release cells or snap freezing and storage prior to single-nuclei extraction can significantly impact the gene expression profiles of cells [43]. Extended times to freezing or processing can negatively affect the data generated from sequenced tissues [Fig. 4]. Significant obstacles need to be addressed in LMICs, including limited local access to tissue processing and sequencing facilities, alongside liquid nitrogen, and a lack of –80 °C freezers. Single-nuclei RNAseq removes the requirement for local facilities to have single-cell library preparation facilities, as samples can be stored and shipped to processing hubs for retrospective studies. However, it still requires access to liquid nitrogen or dry ice for snap freezing and –80° C freezers for storage. A lack of cold storage, more generally, can compromise the stability of RNA and reagents and lead to deterioration due to temperature changes.

Moreover, single-cell technology-based experimental designs and protocols are often expensive, labour-intensive, time-consuming and susceptible to biological noise and technical variation [44]. This challenge arises from several factors, including:

1. *Low RNA content*: Depending on tissue type, each cell averagely contains only ~10 picograms of RNA [45]. Usually, a fraction of this RNA can be captured, affecting the reliability of detecting specific RNA types and the accuracy of sequenced data. Also, depending on the procurement method, time to freezing/processing and tissue type, the quality of extracted RNA may be compromised, yielding low results [46].
2. *Cell Heterogeneity*: Cell capture and isolation, particularly of rare cell types, can make identifying and assigning corresponding gene identities challenging during gene expression analysis. This is primarily due to higher uncertainty in measuring and interpreting single cell types, states, lineages, and pathways, which exhibit a wide range of expression signatures according to their developmental stages.
3. *Technical Variabilities:* Single-cell methods such as SMART-seq2, MARS-seq, droplet-based, and microfluidic-based ScRNA Seq, among others, offer different advantages, disadvantages, and depending on sample type, feasibility, and research questions [47]. Also, with each method comes specialised library preparation protocols, reagents, and varying sequencing platforms such as 10X Genomics, Illumina, PacBio, Oxford Nanopore technologies and others. This contributes to varying sensitivity in capturing mRNA efficiently, detecting low-expressed genes, and transcriptome (full-length versus 5’ and 3’ tagged-based) coverage with challenges in integrating and comparing generated datasets [48, 49]. Additionally, researchers may have to identify and contend with investing in only one method, which may be at a high risk if it does not become the gold standard for scRNA-Seq laboratory approaches.

Though these challenges are generalised for all research teams, the effects are more pronounced for under-resourced research centres due to the additional laboratory and infrastructure challenges. However, rapid progress is being made to address these challenges in developing novel high-throughput approaches such as multiplexing and microfluidics techniques [50] and ultra-low input RNA sequencing [49], among others, to enhance cell isolation and maximise RNA capture efficiency. Emerging techniques and technologies for validating RNA signatures through spatial and temporal sequencing [50, 51] will enable the accurate determination of the temporal location of RNA molecules within tissues to support meaningful biological insights gained from generated scRNA-Seq datasets [52]. As these advancements continue, the costs associated with single-cell methods should decrease significantly.

Continuous efforts to create a transparent, open-source repository on platforms like GitHub and Protocols.io will enhance source codes and protocol sharing and usage in addition to encouraging depositing single-cell datasets from studies on repositories like the HCA Data Portal, the Chan Zuckerberg (CZ) CELL by GENE Discover platforms and more will support the reproducibility of results, the improvement of methodologies, encourage collaboration and educational opportunities for researchers from LMICs that will strength both laboratory and computational skills. Such measures will help to deliver methodological standardisation and ultimately support transcriptomic research in LMICs.

## Ethical considerations

Since the middle of the previous century, much progress has been made concerning the ethical design and conduct of biomedical research, including developing ethical guidelines and principles [53]. Research ethics governance in global health has been significantly strengthened over the years by setting up research ethics committees and ethical boards that oversee the appropriateness of scientific research, particularly on the African continent [54]. Early efforts to capture genetic diversity through the study of genetic underpinnings of malaria by the MalariaGEN and multiple HIV and tuberculosis clinical studies successfully helped pave the way for establishing ethical protocols on the continent and beyond. The birth of the Human Heredity and Health in Africa (H3 Africa) consortium, a partnership between the African Society of Genetics, the US National Institute of Health (NIH), and the UK-based Wellcome Trust in 2010 is the largest collaborative genomic research network across the continent [19]. Their work has improved genomic research expertise, institutional infrastructure, bioinformatics expertise, and resources for young African scientists [55]. Regarding ethically responsible research, it has consolidated protocols for ethical, legal, and social implications (ELSI) through research studies, workshops, and the promotion of inter-member shared experiences and ideas to jointly tackle issues surrounding collaborative projects [55, 56].

Despite the tremendous progress made in the context of ethics governance in LMICs, ethical reviewing challenges can still arise on the ground [57]. This overview, while not an exhaustive list of issues and concerns, contemplates several ethical considerations identified in the relevant literature pertinent to transcriptomic studies. We focus on the dynamics of interactions between researchers and target populations at individual, community, and collaborative levels that could be relevant to single-cell studies in LMICs.

## Individual dynamics

One of the main concerns [57] engaging local communities or populations in research is that often, researchers coming from different social or geographical backgrounds might lack an understanding of the socio-historical and political background that can frame a patient’s attitude to research, leading to distrust and disinterest in taking part in research. Investigating the factors that may lead to hesitancy to participate in single-cell studies will be vital to establishing a study-specific bioethical framework that identifies and analyses the ethical concerns that may arise from a study’s design to its execution [12]. Existing literature suggests paying particular attention to the principle of cultural humility, which involves sensitivity, respect, and open-mindedness toward the cultural uniqueness, beliefs, and experiences about donating blood or body tissues [58].

Another issue is that of informed consent. For LMICs, limited studies provide clear guidelines on the ethical appropriateness of consent models for longitudinal genomic research and biobanking [59]. Though broad consent protocols are increasingly accepted, there are calls for a stronger emphasis on locally appropriate consent forms with content that properly informs and is comprehensibly supplemented with robust data-sharing governance procedures [60]. An issue that single-cell studies should consider is how to appropriately and effectively explain complex information regarding study procedures, data storage, data access, and governance structures to research participants. Methods employed by other biomedical research studies include the use of innovative methods of communication, such as videos, comics, and animations translated into local languages. This could be a way to try and ensure a better understanding amongst the research population of where samples are going, who they will be shared with, what they will be used for, the benefits they may have, and how they can follow up.

## Community dynamics

Community-engaged research is a continuous and iterative process of partnership and mutual empowerment of researchers and communities [61]. It is usually perceived as an essential way for communities to understand their own research and health needs and for researchers to understand the community priorities intending to address them [62]. Significant literature exists on the different engagement models [59–62]. Studies, including transcriptomic studies, committed to conducting research that is appropriate and relevant to the intended communities would be required to invest time, effort, and resources before the onset of the research to develop a continuous and iterative partnership with their target populations and allow for their research to be shaped by the findings of the community engagement process.

Furthermore, researchers working with previously disenfranchised populations may be met with heightened suspicions and sentiments of feeling disproportionately targeted for experimental research. Though beyond the scope of this review, it will be essential to involve communities to seek perspectives on their experiences, needs, and concerns and incorporate these into the ethical framework of a single-cell study.

## Collaborative dynamics

International research collaborations provide a vital route to access funding, resources, and shared expertise to conduct research for many scientists in LMICs [19, 61]. However, collaborative partnerships between local scientists and researchers from countries with strong scientific footing for genomic studies can be characterised by a power imbalance that can lead to tensions [62]. For this review, we focus on the issue of allocation of research funds and resources, as this is one of the main issues advocates of sustainable research capacity have identified as a prominent driver of bias in the representation and prioritisation of needs resulting in the lack of diversity among researchers [60, 62]. Sustainable research capacity includes “building of human capacity, infrastructural capacity building and the sustainable access to funding” [36] and is directly linked to long-term development [12, 57, 63]. The inequitable access to research funds can lead to a form of “helicopter” research, which involves sending samples out of a local community without efforts to set up core facilities or ensure relevant training and resources to bolster locally spearheaded research [64].

Building or expanding local infrastructure capacity enables local scientists to be better positioned to identify and address ethically appropriate research needs of their communities. Also, it can put them on an equal footing to co-create a research agenda with international collaborators that fosters equitable contribution and mutual benefit. Equitable collaborations may alleviate fears of exploitation of local researchers and increase trust by sharing the burdens of collecting, processing, and sequencing samples [65]. This ensures equal access and ownership of intellectual property, co-authorship of publications, and financial and intellectual independence. Studies focusing on international health research collaborations suggest that establishing rules of engagement, research capacity building, and equitable, collaborative partnerships are central to sustainable genomic research and building trust – and these principles must be translated into single-cell transcriptomic studies [65, 66]. Adapting to challenges that may arise and tailoring study designs and implementation after these identified solutions may prove meaningful toward the success of single-cell transcriptomic studies in LMICs.

## Conclusion

The growing enthusiasm in the biomedical sciences, particularly the paradigm shifts in genomic research and biobanking initiatives in LMICs, proves that commitment to sustainable research in the developing world is critical to increasing the output of translatable high-quality genomic research. Aims to ensure that all groups of people are being represented fairly and appropriately is encouraging and is a positive trend.

To address the challenges surrounding inadequate resources and infrastructure management, lack of technical expertise, limited funds, and ethical and legal considerations for single-cell studies, the following steps can be taken:

1. *Investment in training and capacity building:* Encourage international and local cooperation to pool resources, building sustainable infrastructure and training programs. This will equip local researchers with the necessary transferrable skills and increase the growth of new researchers with adequate expertise.
2. *Building local research manufacturing sites*: Advocate for increased funding for single-cell studies, including consideration of LMICs as potential sites for biotechnology companies’ external facilities. This creates opportunities for global competitiveness and facilitates the establishment of distribution hubs with readily available technical support personnel.
3. *Utilisation of open-source tools*: This would considerably scale down costs and increase access to cutting-edge technology.
4. *Strong security protocols and data management:* Protection of patient privacy and confidentiality of patient metadata for biobanks, even in the case of a lack of clear-cut regulations regarding data handling, is key to respecting the dignity of the people involved in the research.
5. *Strengthening of ethical and legal frameworks*: This will provide clarity and guidelines to undertake transcriptomic research that is ethnically appropriate and responsible.

Addressing these challenges will enable the construction of high-quality ancestrally inclusive single-cell reference atlases that will serve as the foundation for future basic research, translational studies, and personalised medicine. Globally representative cellular atlases will ensure the future benefits of these atlases, such as drug discovery, disease susceptibility, severity, and other specific health-related efforts, are tailored to benefit all groups of people.
